# Missouri State Veterinary Medical Association

**Published:** 1894-11

**Authors:** H. B. Piatt

**Affiliations:** Secretary


### MISSOURI STATE VETERINARY MEDICAL ASSOCIATION.

The third annual meeting of the Missouri State Veterinary Medical Association was held at Merchants' Hotel, St. Louis, Mo., on October 12th, 1894.

Dr. T. B. Howard, President, of Nevada, Mo., in the chair. Morning session. There were present, Dr. T. B. Howard, Nevada, Mo.; T. F. Arnold, Lewiston, Mo.; L. M. Klutz, Clinton, Mo.; T. L. Wolf, Kansas City, Mo.; J. B. Black, Kansas City, Mo.

The minutes of previous meeting read and approved.

The following named gentlemen presented themselves for membership and were elected: Drs. A. Rouif, J. M. Philips, A. E. Philips, M. M. McNally, E. N. Farrell, of St. Louis, Mo.; L. E. Booth, of Moberly; T. J. Turner, State Veterinarian of Columbia; T. F. Kierman, Jefferson City; T. E. White, of Sedalia, Mo. Dr. M. R. Trumbower, State Veterinarian of Illinois, was elected an honorary member. Meeting adjourned until 2 P.M.

Meeting again called to order at 2 P.M. Dr. Arnold read an interesting paper on Actinomycosis, and handled the subject very intelligently.

Dr. L. M. Klutz then followed with a paper entitled "Veterinary Science," which was also very interesting.

Dr. M. R. Trumbower then kindly entertained the meeting with a description of the new laws of Illinois relating to contagious diseases, etc.

The following names were presented and duly elected as members: Drs. R. J. Sollberger, C. W. Crowley, H. F. James, A. J. Hamerstein, H. B. Piatt and Chas. Ellis, all of St. Louis, Mo.

Dr. M. H. Schelle, M.D., was elected an honorary member.

Election of officers being in order, the following named gentlemen were duly elected and installed: Dr. T. J. Turner, State Veterinarian of Columbia, Mo., President; Dr. T. F. Arnold, of Lewiston, Vice-President; Dr. F. L. De Wolf, of Kansas City, Mo., Treasurer; and H. B. Piatt, of St. Louis, Mo., Secretary.

No further business, the meeting adjourned, to meet at Moberly, Mo., on the first Wednesday in September, 1895.

H. B. PIATT, *Secretary.*

